# A cautionary tale of virus and disease

**DOI:** 10.1186/1741-7007-8-124

**Published:** 2010-09-27

**Authors:** Robin A Weiss

**Affiliations:** 1 Division of Infection and Immunity, University College London, 46 Cleveland Street, London, W1T 4JF, UK

## Abstract

The recent identification of the gammaretrovirus XMRV and a second gammaretrovirus of a different subtype in chronic fatigue syndrome has aroused much interest, not least among sufferers. However, it remains highly controversial whether the detection of these viruses represents true infection or laboratory artifacts.

## 

A year ago, Lombardi *et al*. [1] reported in *Science *the detection of a retrovirus in 67% of persons suffering from chronic fatigue syndrome (CFS) compared with a 3.7% infection rate in healthy controls. The virus is known as xenotropic murine related retrovirus (XMRV) because its sequence is closely related to, but distinct from, those of well known strains of xenotropic murine leukemia viruses (XMLVs). In CFS blood samples, XMRV was readily detected by PCR amplification of the viral genome, by the expression of viral antigens in infected cells and by virus isolation and propagation in cell culture. These findings have since become controversial, with reports from four independent groups of investigators [2-5] who have failed to find evidence of any association between XMRV infection and CFS. Most recently, to add to the confusion, a new paper [6] reports an association between CFS and a different retrovirus - a report erroneously described in some of the press coverage as confirmation of the original report of Lombardi *et al*.

Many people suffering from CFS greeted the first report [1] with enthusiasm and relief because of the persistent skepticism of physicians about whether CFS is a defined disease with a single cause. If the association of at least two kinds of murine-related retrovirus with the syndrome stands the test of time, it will represent a very important discovery. CSF patients would then be assured of having a recognized infection with the possibility of effective treatment - indeed, some of them are already so convinced they have started treatment with anti-retroviral drugs (first developed against HIV) in the hope of clearing infection and their symptoms. Blood banks would have to consider whether to screen donations for the implicated retroviruses. But before such steps could be justified, it will be essential to perform truly blinded tests on cases and proper controls in several laboratories. Profoundly disappointing as this would be for patients, without such additional studies, laboratory artifacts cannot be ruled out; also, with the signal exceptions of HIV and human T-lymphotrophic virus, the history of retroviral associations with human disease is not encouraging.

## Mouse gammaretroviruses and human disease

XMRV is a gammaretrovirus closely related but not identical to other XMLV strains. These viruses have the curious property that they can infect foreign cells, such as human cells, in culture, but do not re-infect murine cells [7,8]. The term xenotropic was coined by Jay Levy in 1972 to distinguish these viruses from ecotropic MLV, which infects mouse cells but not human cells. There is also a polytropic MLV that can infect the cells of both species, and it is this virus that most closely resembles the retrovirus identified in the latest claim for a retroviral association with CFS [6]. XMLV is carried by most strains of mice in the form of endogenous viral genomes integrated in the chromosomes of the mouse germ line and inherited as Mendelian traits, but that can be activated to emerge as potentially infectious virus particles. The tropism of the viruses (that is, the cells and species they can infect) is largely determined by the cell surface receptors [9] to which the viral envelope glycoproteins bind before the virus gains entry into the cell. Gammaretroviruses like XMRV are capable of crossing to host species of quite different taxa; for instance, a virus of south-east Asian rodents has jumped to gibbons and to koalas [8,10]. So given our frequent proximity to mice, infection of humans by murine retroviruses is not inherently unlikely.

XMRV was first described in humans in a subset of patients with prostate cancer [11]. This subset was homozygous for a mutation in the gene for RNase L, which is activated in response to the type 1 interferons, which are induced by viral infection and activate innate cellular anitviral defenses. RNase L degrades double-stranded RNA and thereby prevents viruses from replicating. It seemed reasonable, therefore, to suppose that people defective for RNAse L might be more susceptible to infection by a murine virus, either as a direct zoonotic transfer from a mouse or from virus already circulating from human to human. However, the association of XMRV with prostate cancer had a number of mystifying features. XMRV appeared to be integrated into human DNA but, curiously, the virus was located in the stromal cells rather than the carcinoma cells in tumor biopsies [11]. Stromal cells express a receptor for XMRV [9], but an independent group that reported XMRV in late-stage prostate tumors [12] detected the virus in the cancer cells themselves rather than the stroma, and found no association with the RNase L polymorphism. As with the postulated association of XMRV with CFS, other groups in Europe could not find the virus in prostate cancers. Thus, the status of XMRV in relation to prostate cancer remains uncertain. Is it present in the stroma or the carcinoma cells? Is there a genuine association with the host polymorphism in RNase L? Is it really present only in North American patients?

Pat Moore, the co-discoverer of two human oncogenic viruses (Kaposi's sarcoma herpesvirus and Merkel skin cancer polyomavirus) points out on the F1000 blog how surprising the CFS findings results reported by Lombardi *et al*. [1] are, if taken at face value. XMRV appears to be present in peripheral blood mononuclear cells (PMBCs) at extraordinarily high viral loads. Practically every cell in stimulated PBMC cultures apparently expresses viral envelope antigen (according to unimodal flow cytometry peaks in each of the five patients analyzed), and the western blot shows a higher level of envelope expression in patients' PBMCs than in the positive control of an experimentally XMRV-infected cell line. If these results are confirmed as specific rather than antigenic cross-reactions, according to my reckoning a higher proportion of PBMCs could be infected in CFS than is seen in any other retroviral infection of humans or animals. Yet only 9 of 18 of these highly antigen-positive patients produced serum antibodies to the envelope. The response of Lombardi *et al*. to Moore's comments was that they had not made it sufficiently clear in their paper [1] that the PBMCs had been cultured for 7 to 14 days in order to amplify the amount of XMRV.

## Ubiquitous murine retroviruses

Investigators who have no special interest in retroviruses may not realize that XMLV is widespread in many laboratories conducting biological or medical research. Numerous human cancer cell lines have been propagated at some stage of their history as xenografts in immunodeficient mice and have acquired XMLV infection from the mouse. Other human cells have acquired the virus through horizontal infection in the laboratory owing to the less stringent containment practiced in non-virological laboratories. XMLV may also be widespread in laboratory reagents. For example, 28 years ago I reported that in my laboratory, 50% of murine hybridomas producing monoclonal antibodies also secreted XMLV. Another example is HotStart Taq polymerase, which has an inhibitory murine monoclonal antibody blocking cold polymerase activity and may therefore contain traces of XMLV. Thus, the opportunities for XMLV sequence contamination are rife, and any claims about the identification of viruses of this family, including XMRV and polytropic MLV, in human clinical samples need to be regarded with some caution.

I would be happy to accept that if XMRV is associated with CFS, so could polytropic MLV be. What I find extraordinary, however, is that only XMRV was found by Lombardi *et al*. [1], whereas only polytropic MLV was detected by Lo *et al*. [6]. Given that the samples analyzed by Lo *et al*. were collected over several years from persons with no contact with each other and widely dispersed across states in New England [6], it does not make epidemiological sense that the two types of CFS-associated MLV segregate according to the laboratory performing the tests.

If the positive results linking XMRV with CFS are not laboratory artifacts, how can we explain the failure of other investigators to replicate the findings? Much discussion (for example, in the chatroom of the BioMed Central journal *Retrovirology *following the negative reports [4,5]) revolves around the suggestion that the discrepancies might be explained by variation in the diagnostic criteria for CFS. It is a syndrome that has a fairly broad spectrum of signs and symptoms and the precise definitions used by the different investigating groups were not identical. But while different inclusion criteria for CFS could explain a difference between, say, a 50% and a 67% detection frequency of XMRV in CFS samples, surely it cannot account for the difference between 0% and 67% found in these reports.

## Rumor viruses

My own skepticism also derives from a strong feeling of *déjà vu*. Following the discovery of reverse transcriptase 40 years ago, several laboratories started looking for retroviruses (or RNA tumor viruses as they were then called) in human tumors, and in 1972 one was discovered in a human cell line derived from a pediatric rhabdomyosarcoma - a tumor of myoblasts. It took 2 years for three independent groups to show that the virus, hailed as the first human RNA tumor virus, was actually a previously unknown xenotropic retrovirus of cats [7]. Indeed, it turned out that the tumor in question had been passed as a xenograft in the brain of a fetal kitten as an immunologically privileged site (nude mice had not yet become available).

Since that time, there has been a long succession of 'rumor' viruses posing as tumor viruses and promulgated as the cause of chronic human diseases; these are reviewed in detail elsewhere [13]. For instance, the proposition that murine mammary tumor virus (a betaretrovirus) is present in human breast cancer has been in circulation since 1972; but to my mind, the evidence is no stronger for simple reiteration. The stimulus for the invitation to write the comprehensive survey of rumor viruses mentioned above [13] was the publication of a retraction of what we imagined was our own discovery of a novel human retrovirus. In 1997 we reported finding an envelope-defective retrovirus genome in patients with rheumatoid arthritis, only to discover 4 years later (and after two groups, in the USA and Sweden, had independently 'confirmed' our findings) that our putative human virus was actually a previously unknown endogenous betaretrovirus of rabbits.

If virus detection resulted from laboratory contamination, why was our virus detected more frequently in clinical disease samples than in the healthy controls? We have no good answer to this question, but I suspect it may simply be that the vials containing clinical specimens tend to be handled more often than a job-lot of healthy blood samples. If the healthy samples had come from strictly derived case-control collections in which the control vials were handled in exactly the same manner, for the same time and with the same frequency as the vials containing the clinical specimens (which has not been done for CFS), we might not have observed the bias towards detection in cases above controls. Our laboratory controls - water specimens interspersed between PBMC DNA extracts - were reassuringly negative. So I raise an eyebrow when investigators declare that contamination is 'out of the question'; once bitten, twice shy.

Much argument circulates among the protagonists and skeptics of XMRV about which PCR primers should be used and the exact conditions of detection. These matters are, of course, important, but it seems to me that if a virus is genuinely present and is detectable by a single round of PCR amplification, it should be confirmable with several alternative sets of primers for different regions of its genome. The recently reported [6] murine retrovirus distinct from XMRV, more closely related to polytropic MLV (should it be called PMRV?), was also detected by single-round PCR; but in contrast to Lombardi *et al*. [1], the authors of this paper have, to date, reported no virus isolation or serological tests.

Various viruses, including herpesviruses and enteroviruses, have been implicated in CFS. The possible involvement of a retrovirus was first mooted 19 years ago [14]. In that paper, human T-lymphotropic virus type II (HTLV-II) was detected in a study remarkably similar to those reported in the new papers [1,6] (which do not cite it). As in the current story, two independent groups, in the USA and UK, were unable to confirm any link between HTLV-II and CFS. The original paper was never retracted and it appears to be largely forgotten. I wonder which papers will be cited 19 years from now.

## SV40 in human tumors

The current controversy is also highly reminiscent of the storm that raged in the 1990s over the presence of simian virus 40 (SV40) in human tumors [15]. This polyomavirus occurs naturally in macaque monkeys and is related to human polyomaviruses BK and JC, both of which infect the majority of the human population. SV40 was discovered in 1960 by Sweet and Hilleman as a contaminant of rhesus monkey kidney cultures used for the propagation of poliovirus vaccine. This led the vaccine manufacturers to switch cell substrates to SV40-free African green monkey kidney cultures, which, more than 20 years later, were in turn found to harbor a simian version of immunodeficiency virus (SIVagm); luckily, however, this strain of SIV does not infect humans. Anyway, the change in vaccine production did not happen until some millions of people had been immunized with poliovirus vaccine stocks prepared in rhesus kidney cells so there was potential exposure on a massive scale. Accurate serological surveys for SV40 infection in humans were obscured by cross-reactions with the human BK and JC viruses (and during the past 4 years, three novel strains of human polyomavirus have been reported).

SV40 is highly oncogenic in new-born rodents but is not known to be oncogenic in monkeys. Likewise, BK and JC viruses can cause cancer in hamsters but not in their natural host, the human. Epidemiological studies were set up to establish whether any increase in cancer occurred in populations exposed to the potentially contaminated poliovirus vaccines, but no excess cancer incidence was found. In 1992, however, SV40 was reported in pediatric cases of neurological tumors, patients whose parents, or more likely grandparents, might have been exposed to the tainted vaccine. Positive sightings of SV40 were soon reported in other human tumors too, especially mesothelioma and osteosarcoma. If these reports could be confirmed, it would imply that SV40 is now circulating as a transmissible virus in humans and that it is oncogenic. Several other investigators, however, could not find evidence of SV40 in tumor biopsies [15].

The protagonists of SV40 in human tumors said the doubters did not know how to conduct proper, specific PCR tests; the skeptics pointed out how easy it is for SV40 to contaminate samples. SV40 sequences are ubiquitous in molecular biology laboratories as they are represented in so many plasmids and reagents. Sasha Voevodin and Preston Marx recently stated that 'The genomic DNA of SV40 is the most intensively manipulated DNA molecule (per base pair) in the history of molecular biology' [10]. MLV must be a close runner-up given the frequency with which we use retroviral vectors derived from them.

The National Cancer Institute and the Food and Drug Administration (FDA) did the right thing for SV40, just as is now proposed by the FDA for XMRV, and sponsored a multi-center study of strictly masked samples of mesothelioma and normal lung tissue, and included positive controls deliberately spiked with the virus [16]. Only the positive controls came through as consistently containing SV40 DNA. Drafting a timely report, however, proved to be far more difficult than performing the tests, owing to back-tracking by some protagonists concerning earlier agreement on the methods and the specimens to be used, and I think that the federal agencies tried too hard to reach a consensus where none existed. The Institute of Medicine later produced a report stating that no conclusive evidence of SV40 transmission to humans from poliovirus vaccines was evident [15]. The proposed XMRV survey of masked samples could benefit from the experience of the SV40 exercise.

## Absence of evidence and evidence of absence

It is noteworthy that positive findings are often published in high profile journals, while the negative ones find their way to specialist journals. In the case of XMRV, two of the negative reports were accepted by *BMC Biology*'s sister journal *Retrovirology *[4,5], which is becoming well established as the standard-bearer of its field. The recent paper by Switzer *et al*. [5], which examined properly blinded samples, perhaps has too modest a title: 'Absence of evidence of XMRV virus infection in persons with CFS and healthy controls in the United States'. I would go further and suggest that they found firm evidence of absence.

Despite the arguments and observations that I have raised, I feel some discomfort as a retrovirus specialist in casting doubt on the link between XMRV and prostate cancer and CFS. During the past two decades I have spent far more time than I wished arguing against those who deny that HIV is the cause of AIDS. Moreover, I have acted as a Jeremiah, warning of the potential hazards of clinical xenotransplantation in case endogenous retroviruses of pigs (gammaretroviruses related to MLV) might cause havoc in humans treated with animal cells or tissues.

In this journal, Martin Raff recently answered questions about the biological basis of autism [17] and repudiated the notion that either infection or MMR immunization is the culprit. The difficulties of CFS are similar to those of autism, precise diagnosis being a major problem; and at the time of writing, the question of murine retrovirus infection in CFS remains open. Rumor viruses are seldom eradicated; they remain latent, waiting to be reactivated in a new disease. Subtle diseases of unknown cause will remain susceptible to rumor viruses for as long as no other etiology is established.
